# Economic and Health Predictors of National Postpartum Depression Prevalence: A Systematic Review, Meta-analysis, and Meta-Regression of 291 Studies from 56 Countries

**DOI:** 10.3389/fpsyt.2017.00248

**Published:** 2018-02-01

**Authors:** Jennifer Hahn-Holbrook, Taylor Cornwell-Hinrichs, Itzel Anaya

**Affiliations:** ^1^Department of Psychology, University of California, Merced, Merced, CA, United States; ^2^Center for Excellence in Biopsychosocial Approaches to Health, Chapman University, Orange, CA, United States; ^3^Department of Health Sciences, Chapman University, Orange, CA, United States; ^4^Department of Psychology, Palo Alto University, Palo Alto, CA, United States

**Keywords:** depression, prevalence, gini index, postpartum, gross domestic product, pregnancy, infant mortality

## Abstract

**Background:**

Postpartum depression (PPD) poses a major global public health challenge. PPD is the most common complication associated with childbirth and exerts harmful effects on children. Although hundreds of PPD studies have been published, we lack accurate global or national PPD prevalence estimates and have no clear account of why PPD appears to vary so dramatically between nations. Accordingly, we conducted a meta-analysis to estimate the global and national prevalence of PPD and a meta-regression to identify economic, health, social, or policy factors associated with national PPD prevalence.

**Methods:**

We conducted a systematic review of all papers reporting PPD prevalence using the Edinburgh Postnatal Depression Scale. PPD prevalence and methods were extracted from each study. Random effects meta-analysis was used to estimate global and national PPD prevalence. To test for country level predictors, we drew on data from UNICEF, WHO, and the World Bank. Random effects meta-regression was used to test national predictors of PPD prevalence.

**Findings:**

291 studies of 296284 women from 56 countries were identified. The global pooled prevalence of PPD was 17.7% (95% confidence interval: 16.6–18.8%), with significant heterogeneity across nations (*Q* = 16,823, *p* = 0.000, *I*^2^ = 98%), ranging from 3% (2–5%) in Singapore to 38% (35–41%) in Chile. Nations with significantly higher rates of income inequality (*R*^2^ = 41%), maternal mortality (*R*^2^ = 19%), infant mortality (*R*^2^ = 16%), or women of childbearing age working ≥40 h a week (*R*^2^ = 31%) have higher rates of PPD. Together, these factors explain 73% of the national variation in PPD prevalence.

**Interpretation:**

The global prevalence of PPD is greater than previously thought and varies dramatically by nation. Disparities in wealth inequality and maternal-child-health factors explain much of the national variation in PPD prevalence.

## Introduction

Maternal mental health problems pose major public health challenges for societies across the globe. For example, psychiatric illness (often associated with suicidality) is one of the leading causes of maternal death in the UK (1), as well as a leading killer of women of childbearing age in both India and China (2). The most common psychiatric malady following childbirth is postpartum depression (PPD), a devastating mental illness that can impair maternal behaviors (3, 4) and adversely affect the cognitive, emotional, and behavioral development of children (5).

Three decades of interdisciplinary research have produced thousands of studies investigating the characteristics, measurement, consequences, treatment, and predictors of PPD. Despite these efforts, the global prevalence of PPD remains unknown. The widely cited PPD prevalence rate of 13% ascertained two-decades ago is based on a meta-analysis of overwhelmingly Western samples (6) and most likely do not reflect the incidence of PPD in the majority of the world’s population. For example, a systematic review and meta-analysis that focused exclusively on low- and lower-middle income countries found a higher incidence of postpartum mental health disorders (7). However, this review, too, did not include wealthy nations for purposes of comparison, leaving open the possibility that the apparently inflated incidence of PPD in the developing world was an artifact of the different study methods employed in those societies (7). For example, low-income countries are more likely than high-income countries to rely on self-report PPD measures (rather than interviews) in the first weeks after birth (7), and we know that self-reported PPD measures taken earlier postpartum tend to yield higher PPD prevalence than interview tools given later. Accordingly, a meta-analysis comparing PPD prevalence, and taking into account divergent research methods used in high-, medium-, and low-income countries, is required to determine the true global and cross-national variation of PPD prevalence.

Further, to our knowledge, no prior large-scale meta-analysis has considered potential cross-national differences in PPD, despite qualitative evidence suggesting that PPD may vary dramatically from nation to nation even between nations of comparable economic standing (8, 9). Reliable national PPD estimates could help to illumine particular economic, health, and policy factors that inflate or reduce PPD prevalence, thereby informing prevention efforts. Further, generating reliable national estimates of PPD could aid policy-makers in decisions about where to allocate limited resources, and alert global health agencies to direct aid to those countries most impacted.

Motivated by the potential health benefits of filling these knowledge gaps, we conducted the largest meta-analysis and meta-regression to date of global PPD prevalence. The present meta-analysis contains four times more studies, 22 times more women, and data from an additional 36 nations compared to the largest previous meta-analysis of PPD prevalence (6). We aimed to estimate PPD prevalence both globally and by nation and to explore whether divergent methodologies or disparities in health, economic, policy, or sociodemographic factors explain cross-national differences in PPD.

## Methods

This study was comprised of three phases: (1) conducting a systematic review in accordance with PRISMA guidelines (10), (2) performing a meta-analyses to estimate PPD prevalence both globally and for each nation, and (3) using meta-regression to investigate whether methodological, economic, health, and/or policy factors predict cross-national variation in PPD.

### Search Strategy and Selection Criteria

To identify potentially eligible articles, we searched PubMed, PsychINFO, and CINAHL using a combination of the following MeSH terms in the abstract: (“postpartum depression” or “postnatal depression”) and (“incidence” or “prevalence”). In addition, we used the measures and instruments qualifier “edinburgh postnatal depression scale.” We further limited our search by only including studies of human females published in English between 1985 (just before the EPDS scale was published) and 2015. The exact Boolean searches used for each database are provided in Section “Boolean Search Information” in the Appendix. Additionally, we reviewed three previously published comprehensive literature reviews of PPD prevalence (7–9).

To be eligible for inclusion in this meta-analysis, studies were required to report PPD prevalence using the Edinburgh Postpartum Depression Scale (a 10-item self-report, widely used tool specially designed to measure PPD; EPDS) (11) on samples of mothers ≤1 year postpartum with a sample size >20. We chose to include studies conducted anytime in the first year postpartum because this is a convention used in the empirical literature (12) [despite the fact that the American Psychological Association categorizes PPD as occurring anytime in the first 4 weeks postpartum (13), whereas PPD is defined as depression occurring anytime within the first 6 weeks by the World Health Organization (14)]. To address the important issue of timing, we examined whether the timing of assessment influenced PPD prevalence through meta-regression in this paper. We also excluded studies reporting PPD prevalence in samples unlikely to be representative of the general population (e.g., studies that exclusively recruited women with a history of depression, teen mothers, immigrant mothers, abused mothers, mothers seeking treatment, mothers of high-risk infants, etc.).

**Figure 1:**
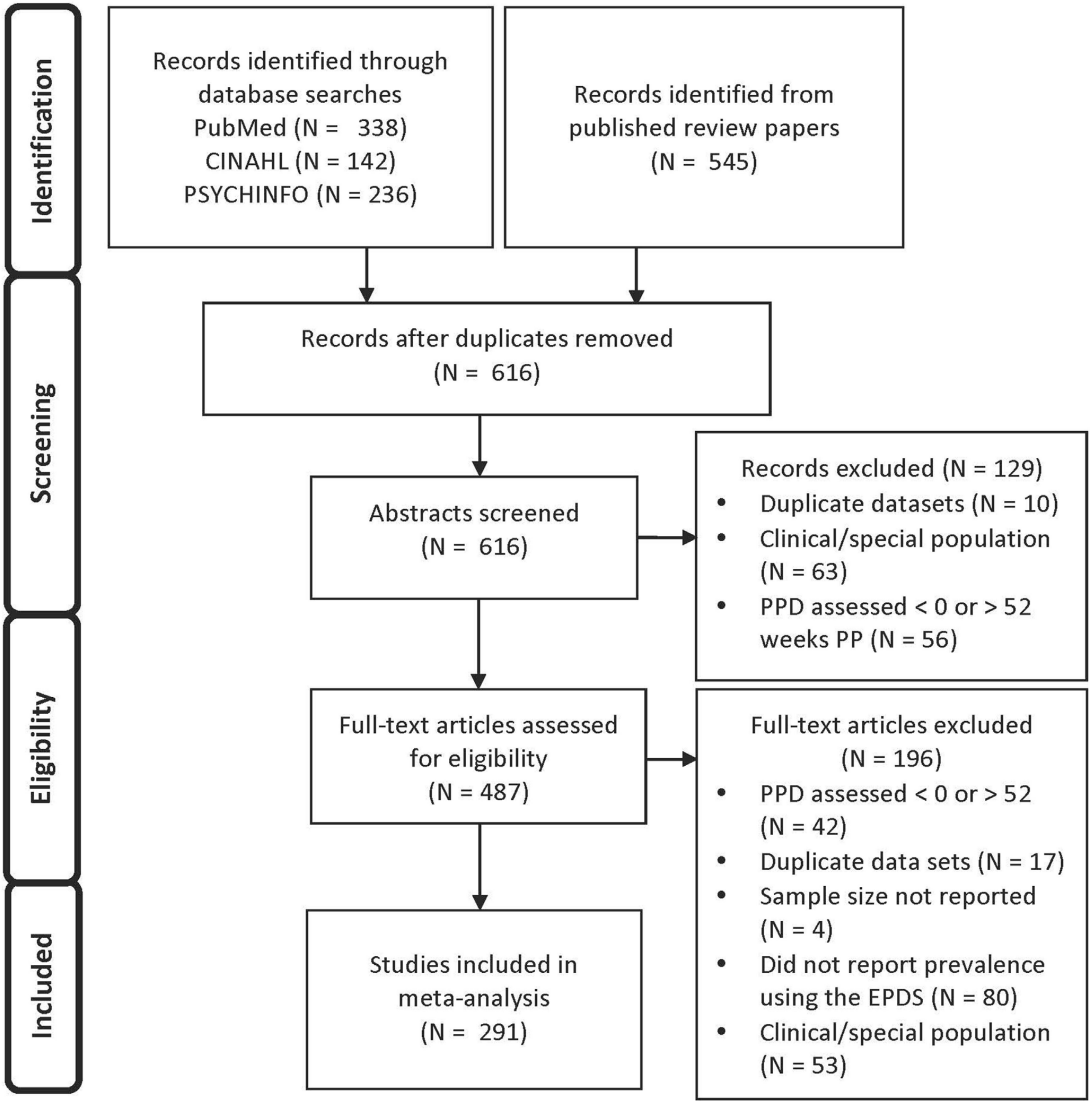
PRISMA flow diagram reporting identification and selection of studies for the meta-analysis.

291 studies (of 487 full-text articles assessed for eligibility) met these criteria and were included in this meta-analysis (see Figure 1 for a PRISMA flow diagram reporting identification and selection of studies for the meta-analysis).

Studies using the EPDS to estimate PPD prevalence were the focus of this meta-analysis and meta-regression for several reasons. First, a recent systematic review of the validated screening tools for common mental disorder strongly recommended the use of the EPDS because it consistently performs well on metrics of internal and external validity, is easy enough to administer in resource-limited settings, and does not include the word “depression” which is stigmatized in some cultures (15). Second, there are advantages to keeping the type of screening tool used consistent across countries when trying to quantify and illuminate the causes of cross-national variability. For example, the wealth of a country strongly determines the type of PPD screening tool used (16) (e.g., it is harder to use time-intensive clinical interviews in resource-poor settings yet easier in resource-rich settings), and the type of screening tool used can influence PPD prevalence (6, 17). Had we included multiple screening tools that differed on ease of administration (e.g., self-report vs. clinical interviews), it would have been difficult to determine whether any observed cross-national variance in PPD prevalence was due to disparities in national wealth or merely an artifact of the assessment tool used. Third, the EPDS had been widely translated and validated for use in at least 18 languages and exhibits good cross-cultural reliability (18). In addition, an examination of previously published systematic reviews showed that roughly 70% of studies used the EPDS to assess PPD prevalence (6, 8, 9). Therefore, the use of the EPDS allowed us to include the majority of studies while limiting confounding variables associated with different types of measurement (8). Finally, because the EPDS is specifically designed for administration in the postpartum period, the scale does not include items assessing changes in appetite, sleep, or weight. Changes in these factors are normal in the postpartum period, yet these somatic items are included as indicators of depression by other self-report screening tools designed to assess depression outside of the postpartum window (e.g., Patient Health Questionnaire-PHQ-9, The Hamilton Rating Scale for Depression-HAM-D, Center for Epidemiologic Studies Depression Scale-CESD, Beck Depression Inventory-BDI, and Zung’s Self-Rating Depression Scale-SDS).

### Data Extraction

The following methodological variables were coded from each study: PPD prevalence, total sample size, EPDS cutoff score employed, and the timeframe postpartum in which PPD was assessed. Because meta-analysis requires one estimate of PPD prevalence per study, data from longitudinal studies reporting PPD in the same women at multiple time points were consolidated by averaging the PPD prevalence over the time points weighted by the sample size at each time point. Also, if multiple prevalence rates were reported in the same study using different EPDS cutoffs, the prevalence rate from the lowest EPDS cutoff was chosen by default. This decision could cause a bias toward higher estimates of PPD incidence; therefore, we also used meta-regression to estimate PPD prevalence at the standard recommended EPDS cutoffs for possible (9/10) and probable (12/13) PPD (11).

To investigate whether studies including women earlier or later in the postpartum period report higher PPD prevalence, we created scores for each study reflecting the range of the timeframes postpartum during which PPD was assessed.

### National Data

Various methodological, health, economic, policy, and sociodemographic variables were explored as potential predictors of cross-national variation in PPD. Potential cross-national predictors of PPD were chosen because they had been previously hypothesized to predict PPD and reliable national data were available for the majority of counties represented in this meta-analysis. See Data Sheet S1 in Supplementary Material for an Excel file containing all of the national data used.

#### Methodological Variables

A previous meta-analysis of PPD suggested that it is important to rule out the possibility that cross-national variation in PPD prevalence is explained by methodological conventions used in different countries (7). For example, it is important to know whether systematic methodological differences like assessing PPD earlier postpartum or using higher/lower EPDS cutoff scores are employed in some countries more often than others. Further, if methodological conventions do differ across countries, we need to know the extent to which these explain the apparent cross-national variation in PPD prevalence. To explore this possibility, country sample-size-weighted national averages for each methodological variable were calculated for use in meta-regression models. In addition, we used meta-regression to assess whether the number of studies conducted in a country predicted cross-national PPD prevalence.

#### Health Variables

Health variables were obtained from UNICEF (19) unless otherwise noted and included infant mortality rate (the probability of dying between birth and age one, expressed per 1,000 live births), lifetime risk of maternal death (the annual number of deaths of women from pregnancy-related causes per 100,000 live births), total fertility rate (the number of children that would be born per woman if she were to live to the end of her childbearing years and bear children in accordance with prevailing age-specific fertility rates), and percentage of low-birthweight infants (born weighing <2,500 g). Percentage of cesarean births was obtained from the World Health Report (20).

#### Economic and Policy Variables

GINI index (an index of the income distribution of a nation’s residents wherein higher values indicate greater wealth inequality) data were obtained from Ortiz and Cummins (21). Gross Domestic Product (GDP) per capita (in adjusted US dollars) and percentage of women working ≥40 h a week (aged 25–30) data were obtained from the Annual labor force statistics (22). Additionally, we investigated national provisions for paid and unpaid maternity leave available from the international labor office (23).

#### Sociodemographic Predictors

The percentage of children living in single parent homes and the percentage of infants born outside of marriage data were obtained from the World Family Map (24). The percentage of urbanized population data were also obtained from UNICEF.

### Data Analysis

Following the recommendations for meta-analysis of prevalence (25), we used a double-arcsine transformation of the PPD prevalence data before calculating the study weights and 95% confidence intervals (CIs) to avoid the undue large weights obtained for studies with low or high prevalence (prevalence close to 0 or 1). To test for heterogeneity in the data, both the Cochran *Q* test statistic and the *I*^2^ statistic were consulted (26). The same procedure was followed to create meta-analytically derived national estimates of PPD prevalence based solely on the studies available from each country. Meta-analytic estimates of PPD prevalence could not be calculated in countries with fewer than two studies (*N* = 16) (27). All meta-analyses were conducted using the program MetaXL and the “prev” command (25).

Two sets of meta-regressions were performed, the first addressing which methodological factors predicted variation in PPD across all studies, regardless of the nation in which the study was conducted, and the second addressing predictors of PPD variation across nations. All meta-regression analyses were performed with STATA 14 (28) using the “metareg” command with random-effects models (because all tests indicated significant heterogeneity). To obtain the SEs needed to weight studies (or nations) for meta-regression in STATA, we transformed the 95%-CIs provided by MetaXL using the following formula (upper 95% CI − lower 95% CI)/3.92. Because national data were not available for all variables, the number of countries included is reported for each meta-regression result using national variables.

Funnel plots, Doi plot analysis, and the LFK index were used to assess potential publication bias. Specifically, to test whether papers are more or less likely to be published due to higher/lower PPD prevalence.

Statistical significance was evaluated using 2-tailed 0.05-level tests.

## Results

### Meta-Analysis of Global PPD Prevalence

**Figure 2:**
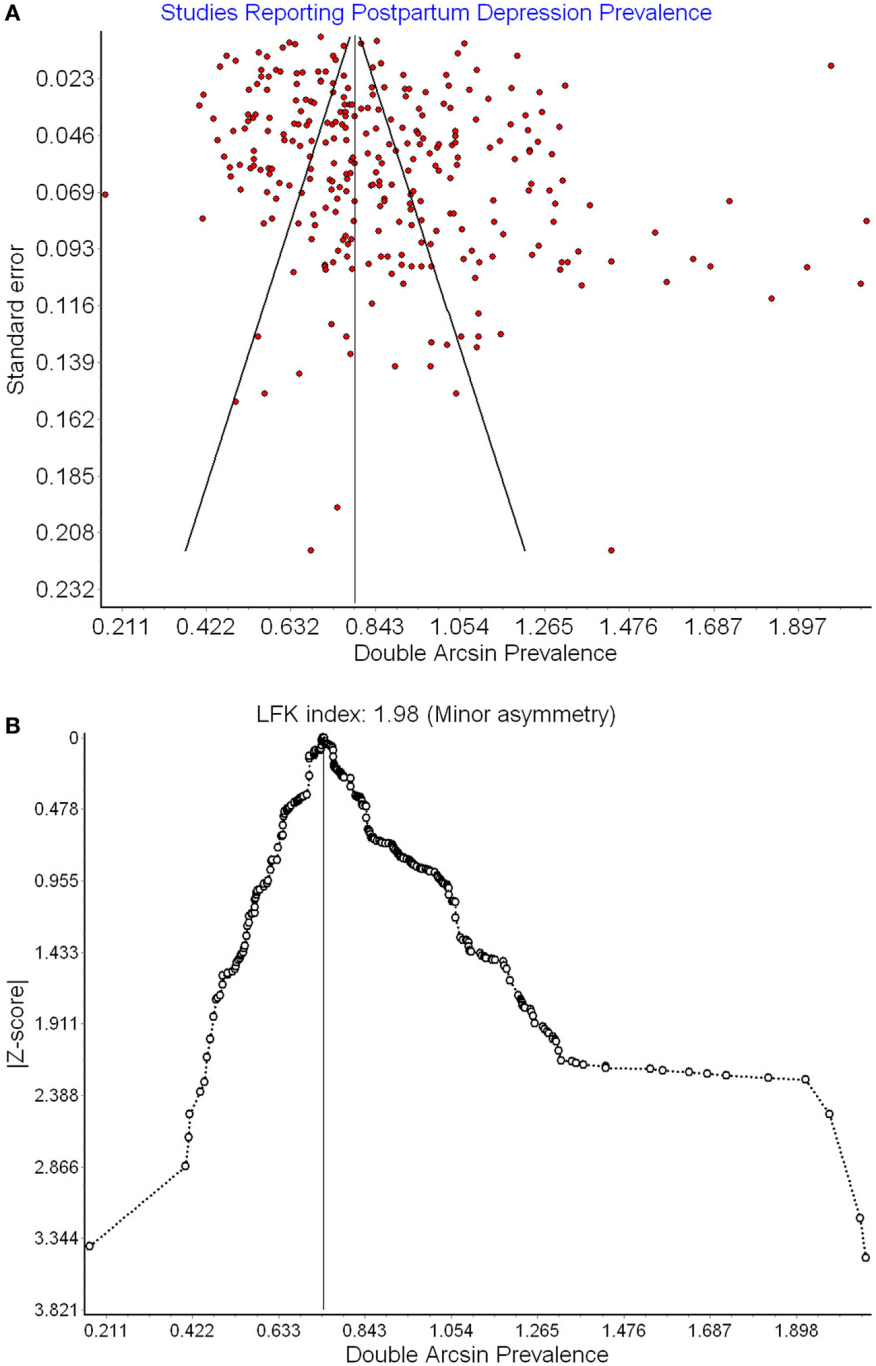
Funnel plot **(A)** and Doi plot **(B)** of postpartum depression (PPD) prevalence as a function of prevalence estimate SE.

296,284 women from 291 studies were included in this meta-analysis. Table 1 presents the data extracted from each study. The global pooled prevalence of PPD was 17.7% (95% CI: 16.6 to 18.8%; see Figure S1 in Supplementary Material). There was a significant degree of heterogeneity between studies (*Q* = 16,823, *p* = 0.000, *I*^2^ = 98%). Adjusting for the recommended EPDS cutoffs yielded a global PPD prevalence of 21.0% (CI: 19.1 to 23.0%) for possible PPD and 16.7% (CI: 14.9 to 18.6%) for probable PPD. See Figure S1 in Supplementary Material for meta-analytically derived PPD estimates for each individual study. There was evidence of publication bias based on sample size (LFK = 1.98; see Funnel Plot in Figure 2).

**Table 1:** Studies included in meta-analysis.

Reference	*n*	Depression prevalence (%)	Cut-off used	Postpartum assessment (weeks)	Country
Affonso et al. (29)	102	15.8	10	1–6	Australia
Alcorn et al. (30)	866	14.4	12	4–24	
Armstrong et al. (31)	114	26.4	12		
Astbury et al. (32)	790	15.4	13	32–36	
Bilszta et al. (33)	1,966	7.6	13	6–8	
Boyce and Hickey (34)	425	9.1	12	6–24	
Boyce et al (35)	103	12.7	13	4–6	
Brooks et al. (36)	3,853	6.0	13	1–52	
Brown and Lumley (37)	1,331	19.6	13	4–6	
Buist et al. (38)	12,361	15.5	10	6–8	
Condon and Corkindale (39)	212	6.1	13	4–6	
Eastwood et al. (40)	25,455	12.0	10	1–12	
Eastwood et al. (41)	15,389	16.9	10	2–3	
Edwards et al. (42)	421	29.7	10		
Griepsma et al. (43)	185	57.8	13	12	
Leigh and Milgrom (44)	161	11.2	13	10–12	
Maloney (45)	399	18.0	13	4–6	
Miller et al. (46)	325	25.0	9	6–24	
Stamp and Crowther (47)	222	9.4	13	6–24	
Stamp et al. (48)	108	17.0	13	4–6	
White et al. (49)	316	20.3	10	6–52	
Willinck and Cotton (50)	358	7.0	13	6–8	
Wynter et al. (51)	172	12.2	9	24	

Kohl et al. (52)	95	9.5	12	1	Austria

Al Dallal and Grant (53)	237	37.1	12	8	Bahrain

Edhborg et al. (54)	674	14.0	10	8–12	Bangladesh
Gausia et al. (55)	346	22.0	10	6–8	

Da-Silva et al. (56)	21	42.8	13	4	Brazil
de Almeida et al. (57)	222	16.2	13		
Filha et al. (58)	12,764	25.8	13	24–36	
Lobato et al. (59)	811	24.3	12	0–20	
Lobato et al. (60)	456	24.8	12	6–8	
Matijasevich et al. (61)	4,109	13.3	13	12–52	
Melo et al. (62)	555	10.8	12	4–6	
Morais et al. (63)	87	19.1	12	16–52	
Pinheiro et al. (64)	207	20.3	13	6–12	
Silva et al. (65)	1,109	16.5	13	4–8	

Bernazzani et al. (66)	213	12.7	13	24	Canada
Bowen et al. (67)	649	8.1	12	4	
DaCosta et al. (68)	78	63.0	12	4–38	
Dennis and Letourneau (69)	498	8.0	13	8	
Dennis and Ross (70)	425	14.1	10	8	
Dennis and Vigod (71)	497	20.7	10	8	
Dennis et al. (72)	498	24.8	10	1–8	
Dennis et al. (73)	315	7.0	13	12	
Malta et al. (74)	972	10.0	10	16	
McDonald et al. (75)	1,578	13.0	10	16	
Sword et al. (76)	2,560	7.6	12	6	
Verreault et al. (77)	226	16.4	10	12	
Vigod et al. (78)	6,126	7.5	13		

Florenzano et al. (79)	88	50.0		0–2	Chile
Jadresic et al. (80)	108	28.7	10	8–12	
Jadresic et al. (81)	542	36.7		8–12	
Risco et al. (82)	103	37.6		1–12	

Gao et al. (83)	130	13.8	13	6–8	China
Gao et al. (84)	126	14.3	13	6–8	
Leung et al. (85)	694	7.2	10	6	
Xie et al. (86)	300	17.3	13	6	
Xie et al. (87)	534	19.3	13	2	

Nielsen Forman et al. (88)	5,091	5.5	13	6	Denmark

Affonso et al. (29)	58	21.8	10	1–6	Finland
Hiltunen et al. (89)	185	14.7	13	1–36	
Luoma et al. (90)	147	10.0	13	8	

de Tychey et al. (91)	277	11.1	12	4–8	France
Frossey et al. (93)	126	11.0	12	1	
Gaillard et al. (94)	264	16.7	12	6–8	
Glangeaud-Freudenthal and Kaminski (95)	604	11.0	13	8	
Guendeney and Fermanian (96)	87	73.5	11	16	
Sutter-Dallay et al. (97)	497	5.8	12	6	

Ballestrem et al. (98)	772	17.0	10	6–8	Germany
Bergant et al. (99)	110	19.0	10	1	
Mehta et al. (100)	419	11.2	9	1–32	
Reck et al. (101)	891	23.6	10	2–6	
Zaers et al. (102)	50	21.7	10	6–24	

Chatzi et al. (103)	529	14.0	13	8–10	Greece
Gonidakis et al. (104)	402	19.8	12	1–24	
Koutra et al. (105)	438	13.0	13	8	
Lambrinoudaki et al. (106)	57	23.5	11	1–6	
Thorpe et al. (107)	165	13.0	12	4	

Affonso et al. (29)	106	53.3	10	1–6	Guyana

Lau and Chan (108)	1,200	34.4	9	1	Hong Kong
Lee et al. (109)	145	11.3	13	6
Lee et al. (110)	244	24.2	10	6
Leung et al. (111)	269	19.8	13	6
Tiwari et al. (112)	3,036	69.9	10	1

Nagy et al. (113)	988	10.8	13	3–26	Hungary

Thome (114)	734	14.0	13	8–12	Iceland

Affonso et al. (29)	110	33.4	10	1–6	India
Dubey et al. (92)	293	6.1	10	1	
Ghosh and Goswami (115)	6,000	25.08	13	1	
Jain et al. (116)	1,537	7.31	12	1	
Mariam and Srinivasan (117)	132	30.0	12	6–10	
Patel et al. (118)	134	48.5	11	1	
Patel et al. (119)	270	23.0	12	6–24	

Andajani-Sutjahjo et al. (120)	274	7.4	12	6–24	Indonesia

Abbasi et al. (121)	416	34.1	13	12	Iran
Abdollahi et al. (122)	2,083	19.4	12	8	
Goshtasebi et al. (123)	281	5.5	13	4–6	
Kheirabadi and Maracy (124)	1,291	26.3	14	6–8	
Montazeri et al. (125)	100	20.0	13	6–14	

Ahmed et al. (126)	1,000	28.4	10	6–8	Iraq

Crotty and Sheehan (127)	625	27.0	12	6	Ireland
Cryan et al. (128)	377	28.6	13	1–52	
Lane et al. (129)	242	11.0	13	6	
Leahy-Warren et al. (130)	410	12.3	11	6	

Alfayumi-Zeadna et al. (131)	564	31.0	10	4–28	Israel
Bloch et al. (132)	210	33.0	10	1	
Bloch et al. (133)	1,286	6.8	10	1	
Dankner et al. (134)	327	11.0	9	6–10	
Eilat-Tsanani et al. (135)	574	9.9	13	8	
Fisch et al. (136)	327	5.2	13	6–12	
Glasser et al. (137)	288	22.6	10	6	
Glasser et al. (138)	104	43.0	10	1–36	

Affonso et al. (29)	100	55.5	10	1–6	Italy
Benvenuti et al. (139)	113	38.9	13	8–12	
Carpiniello et al. (140)	61	29.5	10	4–6	
Elisei et al. (141)	54	13.9	13.00	1–12	
Giardinelli et al. (142)	590	13.2	10	12	
Gorman et al. (143)	21	9.5	13	24	
Grussu and Quantraro (144)	297	13.0	9	6–8	
Mauri et al. (145)	751	10.4	13	4–52	
Oppo et al. (146)	600	6.7	13	4–24	

Matsumoto (147)	675	14.8	9	12	Japan
Miyake et al. (148)	865	14.0	9	8–36	
Nishigori et al. (149)	677	21.3	9	24–36	
Nishizono-Maher et al. (150)	1,048	13.9	9	12–16	
Ohoka et al. (151)	388	10.3	9	4	
Shimizu et al. (152)	65	12.3	9	4–16	
Tamaki et al. (153)	627	18.2	13	4	
Ueda et al. (154)	70	27.0	9	1–52	
Watanabe et al. (155)	235	12.8	9	1–12	
Yamashita et al. (156)	75	16.0	9	4	

Affonso et al. (29)	97	36.7	10	1–6	Korea
Bang (157)	137	22.6		4	
Kim et al. (158)	239	12.6	10	6	

Chaaya et al. (159)	396	21.0	13	12–20	Lebanon
El-Hachem et al. (160)	228	33.3	9	1	

Dow et al. (161)	154	8.1	13	10–14	Malawi

Azidah et al. (162)	377	22.8	12	1	Malaysia
Kadir et al. (163)	293	24.9	12	1–6	
Kit et al. (164)	154	3.9	14	6	
Yusuf et al. (165)	1,362	14.3	12	1–24	

Felice et al. (166)	229	8.7		8	Malta

deCastro et al. (167)	298	14.8	13	1–36	Mexico
Flores-Quijano et al. (168)	163	24.5	13	2–12	

Agoub et al. (169)	144	20.1	12	2–3	Morocco
Alami et al. (170)	100	21.0	12	0–36	

Dørheim Ho-Yen et al. (171)	426	4.9	13	5–10	Nepal
Regmi et al. (172)	100	12.0	13	8–12	

Blom et al. (173)	4,941	8.0	12	8	Netherlands
Verkerk et al. (174)	277	8.2	12	12–52	

Abbott and Williams (175)	1,376	16.4	13	6	New Zealand
Holt (176)	121	14.0	13	6	
McGill et al. (177)	1,330	20.0	12	24–36	
Webster et al. (178)	206	7.8	13	4	

Abiodun (179)	360	18.6	9	6	Nigeria
Adewuya et al. (180)	478	20.9	13	0–8	
Adewuya et al. (181)	876	14.6	10	6	
Bakare et al. (182)	408	24.8	9	1–52	

Dørheim et al. (183)	2,791	16.5	10	7	Norway
Dørheim et al. (184)	2,088	23.9	10	8	
Eberhand-Gran et al. (185)	56	26.8	10	6	
Eberhand-Gran et al. (186)	2,370	8.9	10	6	
Eberhard-Gran et al. (187)	473	9.1	10	1–52	
Glavin et al. (188)	2,227	10.1	10	6	
Haga et al. (189)	737	13.6	10	6–26	
Markhus et al. (190)	43	6.9	10	13	
Nordeng et al. (191)	1,984	8.1	13	17	

Ahmad and Khan (192)	876	14.6	9	6	Pakistan
Husain et al. (193)	149	36.0	12	12	

Ayoub (194)	235	17.0	10	2–12	Palestine

Duedek et al. (195)	344	16.0	13	6–12	Poland

Augusto et al. (196)	588	12.5	13	8–20	Portugal
Figueiredo and Conde (197)	260	14.4	10	0–12	
Figueiredo and Costa (198)	91	26.7	10	13	
Figueiredo et al. (199)	108	17.6	13	8–12	
Gorman et al. (143)	48	9.5	13	24	

Chee et al. (200)	278	6.8	7	6	Singapore
Kok et al. (201)	200	0.5	16	12	

Lawrie et al. (202)	180	36.2	12	6	South Africa
Lawrie et al. (203)	103	36.9	13	6	

Escriba-Aguir and Artazcoz (204)	420	9.8	11	12–52	Spain
Garcia-Esteve et al. (205)	1,201	21.7	9	6	
Sebastián Romero et al. (206)	190	13.2	12	6–8	

Affonso et al. (29)	108	13.9	13	1–6	Sweden
Agnafors et al. (207)	1,707	12.0	10	12	
Bågedahl-Strindlund and Börjesson (208)	309	14.5	13	12	
Josefsson et al. (209)	1,192	13.0	10	6–8	
Lundh and Gyllang (210)	258	8.0	10	6	
Rubertsson et al. (211)	2,430	12.4	13	8–52	
Seimyr et al. (212)	326	14.6	10	8–52	
Sylven et al. (213)	2,318	10.6	12	1–24	
Wickberg and Hwang (214)	1,655	12.0	12	8	

Burgut et al. (215)	1,379	17.6	12	1–24	Qatar

Alharbi and Abdulghani (216)	352	33.2	10	8–12	Saudi Arabia
Al-Modayfer et al. (217)	571	13.7	13	5	

Gorman et al. (143)	60	6.7	13	24	Switzerland
Gürber et al. (218)	219	13.4	10	1–3	
Righetti-Veltema et al. (219)	570	10.2	13	12	

Affonso et al. (29)	99	67.3	10	1–6	Taiwan
Chen et al. (220)	226	18.2	10	4–24	
Chien et al. (221)	190	8.4	10	1–52	
Heh et al. (222)	186	21.0	10	4	
Heh et al. (223)	400	23.0	10	4	
Huang and Mathers (224)	101	19.0	13	24	
Huang and Mathers (225)	106	25.5	13	24	
Lee et al. (226)	60	25.0	14	5–8	
Teng et al. (227)	203	10.3	13	6	
Tsao et al. (228)	162	24.1	13	6	

Limlomwongse and Liabsuetrakul (229)	525	16.8	10	6–8	Thailand

Akman et al. (230)	60	13.6	13	4	Turkey
Alkar and Gençöz (231)	151	74.0	10	1	
Aydin et al. (232)	728	34.6	13	0–52	
Aydin et al.(233)	341	35.8	12.5	0–52	
Ayvaz et al. (234)	152	21.1	13	6–24	
Bugdayci et al. (235)	1,447	37.4	13	0–52	
Danaci et al. (236)	257	14.0	13	4–24	
Dindar and Erdogan (237)	679	32.7	12	8–52	
Ege et al. (238)	364	33.2	13	6–48	
Ekuklu et al. (239)	178	40.4	12	6	
Goker et al. (240)	318	31.4	13	6	
Gulseren et al. (241)	125	13.6		5–26	
Inandi et al. (242)	2,514	27.2	13	1–52	
Inandi et al. (243)	1,350	31.1	13	1–52	
Kirpinar et al. (244)	479	15.9	13	1–6	
Orhon et al. (245)	103	27.2	12	4	
Poçan et al. (246)	187	28.9	13	4–6	
Tezel and Gözüm (247)	567	12.9	11	1	
Yagmur and Ulukoca (248)	785	21.0	13	1–52	

Ghubash and Abou-Saleh (249)	94	18.0	12	1	United Arab Emirates
Green et al. (250)	86	39.4	10	12–24	
Hamdan and Tamim (251)	137	16.8	10	8	

Brugha et al. (252)	190	17.4	11	12	United Kingdom
Cooper et al. (253)	5,124	31.8	9	6–8	
Edge et al. (254)	301	32.0	12	6	
Evans et al. (255)	9,028	9.1	13	8	
Hearn et al. (256)	176	17.0	12	7	
Heron et al. (257)	207	14.0	13	1–8	
Honey et al. (258)	223	17.0	13	6	
Huang and Mathers (224)	50	18.0	13	12	
Matijasevich et al. (61)	13,798	9.6	13	8–24	
Morrell et al. (259)	3,449	17.3	12	6	
O’Higgins et al. (260)	2,048	13.9	13	4	
Ramchandani et al. (261)	11,833	10.0	12	8	
Shelton and Herrick (262)	394	24.4	10	1–52	
Thompson et al. (263)	149	18.8	13	12	
Thorpe et al. (107)	101	12.0	12	4	
Warner et al. (264)	2,375	11.8	13	6–8	

Abbasi et al. (265)	2,972	5.1	12	4	United States of America
Affonso et al. (29)	119	34.1	10	1–6	
Beck and Gable (266)	150	14.6	12	2–12	
Birkeland et al. (267)	149	29.0	13	8–52	
Certain et al. (268)	1,519	10.1	13		
Chaudron et al. (269)	60	27.0	10	0–52	
Dagher and Shenassa (270)	526	6.5		8	
Dagher et al. (271)	638	4.7	13	11	
Demissie et al. (272)	652	7.0	13	12	
Doering Runquist et al. (273)	43	24.3	13	4–24	
Eisenach et al. (274)	939	11.2	13	8	
Gaffney et al. (275)	1,447	24.1	10	8	
Georgiopoulos et al. (276)	909	11.4	12	6	
Glynn and Sandman (277)	170	20.0	10	12	
Gorman et al. (143)	41	5.0	13	24	
Hahn-Holbrook et al. (278)	200	20.0	10	13	
Herring et al. (279)	850	4.0	13	24	
Horowitz (280)	1,071	19.7	10	2–4	
Horowitz et al. (281)	5,169	13.0	10	4	
Howell et al. (282)	242	14.1	13	12–24	
Howell et al. (283)	251	5.5	10	3–24	
Hunker et al. (284)	123	21.0	9	2	
Kim et al. (285)	324	17.0	10	1	
Kuo et al. (286)	139	25.4	13	1–24	
McGrath et al. (287)	114	13.1	12	9–24	
Mercier et al. (288)	688	6.7	13	12–52	
Miller et al. (46)	280	8.0	13	0–16	
Morris-Rush et al. (289)	121	22.0	10	6	
Mosack and Shore (290)	98	14.3	12	24	
Mott et al. (291)	147	7.5	13	52	
Murphy et al. (292)	97	12.0	9	4–6	
Park et al. (293)	25	12.0	13	2–14	
Paul et al. (294)	1,123	4.2	12	1–24	
Reighard and Evans (295)	181	19.9	12		
Rich-Edwards et al. (296)	1,278	8.0	13	24	
Roy et al. (297)	185	17.4	12	6	
Schaper et al. (298)	287	7.0	13	24	
Silverman and Loudon (299)	439	21.4	9	6	
Watkins et al. (300)	2,586	8.6	13	8	
Wisner et al. (301)	10,000	14.0	10	4–6	
Yim et al. (302)	100	22.0	10	8	
Yonkers et al. (303)	802	16.0	12	4	

Chibanda et al. (304)	210	35.5	12	6–7	Zimbabwe

Fisher et al. (305)	506	33.0	12	6	Vietnam

### Meta-Regression of Between-Study Variation

Studies that used lower cutoffs of the EPDS reported significantly higher prevalence (Coef. = −1.44, SE = 0.455, *p* = 0.002; CI: −2.333 to −0.542, *R*^2^ = 3.08%). Studies that measured PPD later postpartum tended to report slightly lower levels of PPD (Coef. = −0.373, SE = 0.109, *p* = 0.001, 95% CI: −0.587 to −0.159, *R*^2^ = 3.65%). No other methodological variables predicted between-study variation in PPD. Together timing of PPD assessment and cutoff used accounted for 5.21% of the variance in PPD prevalence between studies [*F*(2, 293) = 6.44, *p* < 0.002].

### Meta-Analyses of National PPD Prevalence

**Figure 3:**
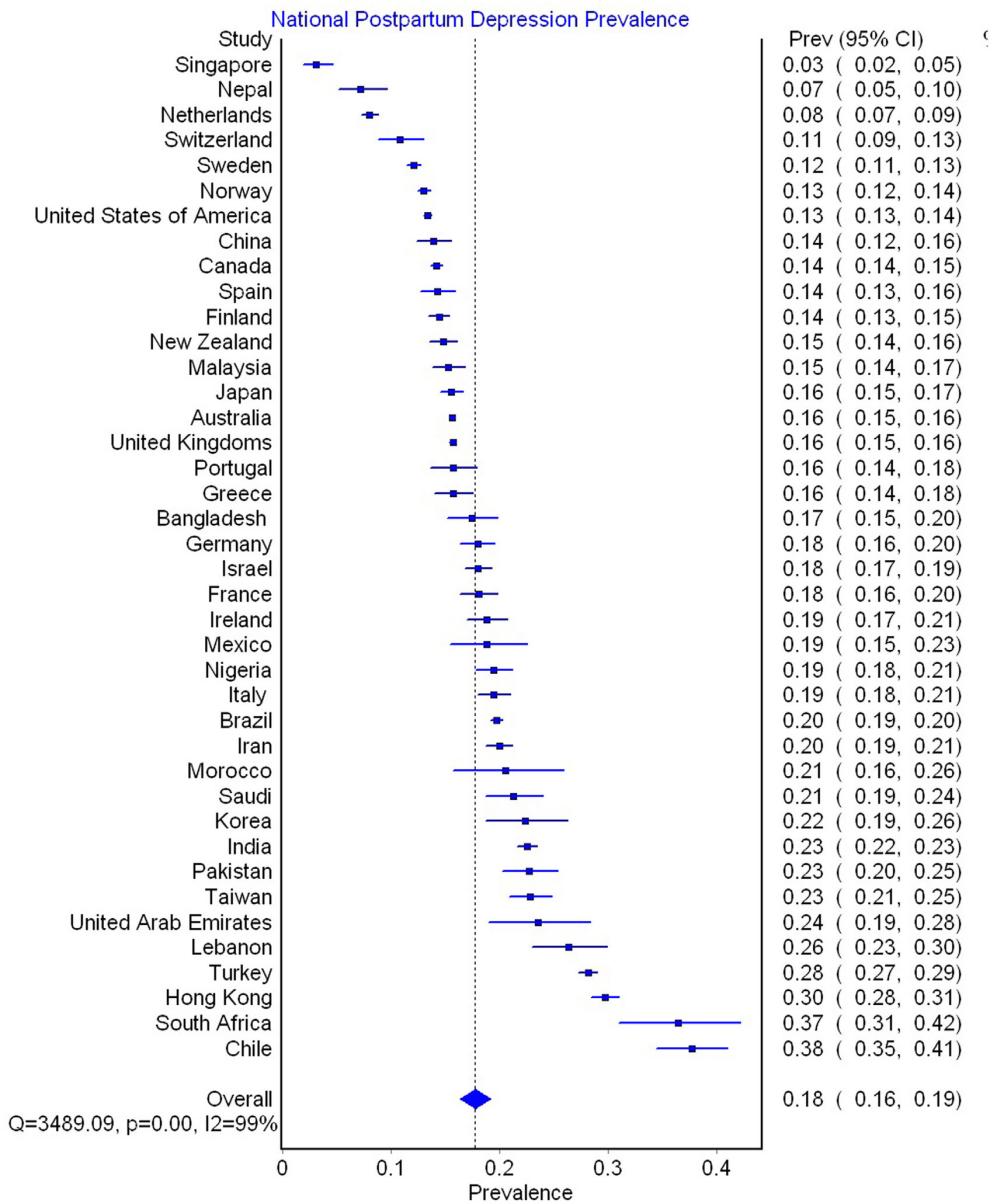
Meta-analytically derived postpartum depression prevalence in 40 Countries.

See Figure 3 for meta-analytically derived estimates of PPD prevalence in 40 countries. National sample sizes ranged from 244 to 65,634 women (M = 7,229.76; SD = 13,502.69). National estimates of PPD ranged from 3.1% in Singapore to 37.7% in Chile. Meta-analysis suggested that there was significant heterogeneity in PPD prevalence between nations (*Q* = 3,489.09, *p* < 0.001, *I*^2^ = 99%).

### Meta-Regression of Predictors of Cross-National Variation

#### Methodological Predictors

None of the methodological variables predicted cross-national variation in PPD prevalence (all *p*s > 0.15). Therefore, no methodological variables were included as covariates in subsequent models.

#### Economic and Policy Predictors

**Figure 4:**
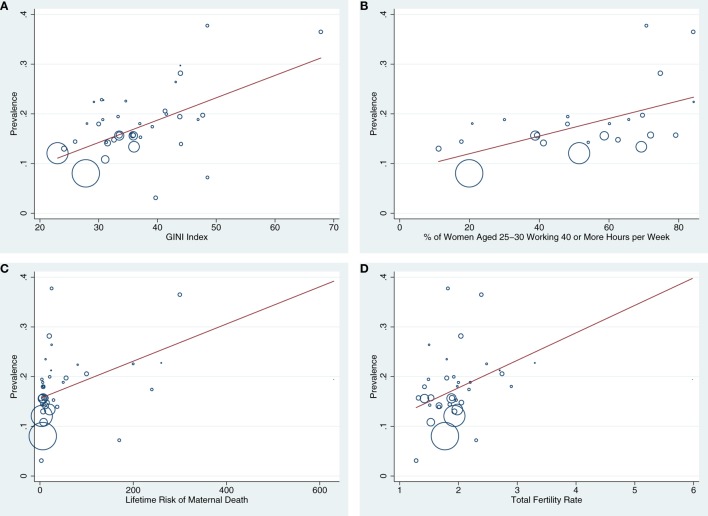
**(A–D)** Bubble plots are presented showing the associations between GINI index **(A)**, % of women aged 25–30 working ≥40 h a week **(B)**, lifetime risk of maternal death **(C)**, and total fertility rate **(D)** with national postpartum depression (PPD) prevalence. Countries with larger bubbles had larger sample sizes and were weighted accordingly in meta-regression models.

GINI index explained 41% of the cross-national variation in PPD prevalence. Nations with higher wealth inequality had higher levels of PPD (*N* = 38; Coef. = 0.039, SE = 0.009, *p* < 0.000, CI: 0.020 to 0.058) (see Figure 4A). GDP per capita was also inversely related to PPD prevalence (*N* = 39; Coef. = −0.033, SE = 0.009, *p* = 0.002, CI: −0.053 to −0.014, *R*^2^ = 30.4%). However, when GDP per capital and GINI index were modeled together, GINI index remained statistically significant while GDP per capita did not. In addition, countries with higher percentages of young women who were working ≥40 h a week had higher PPD prevalence (*N* = 24; Coef. = 0.038, SE = 0.013, *p* < .01, CI: 0.012 to 0.065, *R*^2^ = 30.9%; see Figure 4B). National paid and unpaid maternity leave policies did not predict PPD prevalence (*p*s > 0.60). Together, economic predictors (GINI index, GDP per capita, and women working >40 h per week) accounted for 73.1% of the cross-national variation in PPD prevalence, although GINI index was the only unique economic predictor in a multivariate model.

#### Health Predictors

Rates of maternal mortality and total fertility in Nigeria were more than 4 SDs above the mean, therefore Nigeria was excluded from analyses involving these factors. Higher prevalence of PPD was reported in countries with higher risk of maternal or infant mortality (maternal mortality: *N* = 36; Coeff. = 0.045, SE = 0.019, *p* = 0.024, CI = 0.006 to 0.085), *R*^2^ change = 18.73%, see Figure 4C; infant mortality: *N* = 36; Coeff. = 0.039, SE = 0.018, *p* = *0.034*, CI: 0.003 to 0.074; *R*^2^ change = 15.56%). There were also statistical trends suggesting that higher national PPD prevalence was associated with higher total fertility rates (*N* = 36; Coeff. = 0.040, SE = 0.024, *p* = 0.102, CI: −0.008 to 0.088; *R*^2^ change = 6.33%, see Figure 4D) and higher percentages of infants born low birth weight (*N* = 36; Coeff. = 0.023, SE = 0.014, *p* = 0.094, CI: −0.004 to 0.051; *R*^2^ change = 9.99%). National cesarean rates did not predict PPD prevalence. Together, these health factors predicted 26.03% of the variance in PPD prevalence, although maternal mortality rate was the only unique predictor in multivariate models when all health variables were included.

#### Sociodemographic Predictors

The percentages of infants born outside of marriage, living in single parent homes or in urbanized areas did not predict cross-national PPD prevalence.

In sum, economic and health variables explained 73.87% percent of the cross-national variation in PPD [*N* = 24; *F*(3, 20) = 13.27, *p* < 0.001]. Notably, GINI index was the only significant independent predictor of cross-national PPD incidence when all health and economic predictors were included together in the model.

## Discussion

In the largest meta-analysis and meta-regression of PPD to date, the global prevalence of PPD was found to be approximately 17.7% (95% CI: 16.6–18.8%). Adjusting for the recommended cutoffs provided by the EPDS for possible (≥10) and probable depression (≥13) yielded prevalence estimates of 21.3 and 16.7%, respectively. These estimates are significantly higher than the widely cited prevalence of 13% (95% CI: 12.3–13.4%), derived from a meta-analysis of studies from developed countries (6). Our estimate is more similar to the 19% prevalence for PPD derived from studies of relatively low- and middle-income countries (7). We found some evidence of publication bias wherein larger studies reported lower PPD prevalence (*R*^2^ = 0.8%). However, this effect was small and most likely a byproduct of the fact that countries with more wealth inequality tend to produce studies with smaller sample sizes and wealth inequality (GINI index) between nations predicted 41% of the cross-national variation in PPD in this meta-analysis and meta-regression.

The current meta-analysis also revealed large disparities in PPD prevalence across nations. The countries with the highest rates of PPD were Chile (38%, 95% CI: 35–41%), South Africa (37%; 95% CI: 31–42%), Hong Kong (30%, CI: 28–31%), and Turkey (28%, CI: 27–29%). In contrast, countries with the lowest rates included Singapore (3%; 95% CI: 2–5%), Nepal (7%; 95% CI: 5–10%), the Netherlands (8%; 95% CI: 7–9%), and Switzerland (11%; 95% CI: 7–13%). Surprisingly, these national differences in PPD prevalence could not be explained by methodological conventions used in different counties, for example, the typical EPDS cutoff used, sample size, or the timing of PPD assessment. Instead, the vast majority (73%) of the cross-national variation in PPD prevalence could be explained by economic and health disparities between nations.

Notably, national disparities in PPD appear to exist even among countries that fall within similar economic strata. For example, Chile evinced the highest rates of PPD whereas another high-income nation, the Netherlands, had among the lowest. As many scholars have pointed out (306–308), aggregate wealth metrics like GDP give only a very limited picture of the circumstances of large portions of the population. Instead, we found that wealth disparities (i.e., GINI coefficients) was the most robust predictor of cross-national variation in PPD. Countries with higher GINI coefficients have a greater proportion of citizens living in abject poverty, which is a potent predictor of many mental and physical health problems (309). As previous investigators have also noted, living below the material standards of one’s society equates to possessing low social status—regardless of objective income—which can limit access to less tangible resources like education, opportunity, and security (308). Loss of these forms of social capital is thought to contribute to family dysfunction, health problems, and mood disorders (28).

Relatedly, countries with higher rates of wealth inequality in this meta-analysis also tended to have a higher percentage of women of childbearing age working full-time (Coef. = 0.553, SE = 0.126, *p* = 0.001, CI: = 0.250 to 856, *R*^2^ = 36.9%). This fact may partially explain why countries in which higher proportions of women of childbearing age work full-time have a higher prevalence of PPD. Working full-time while caring for young children can place multiple demands on new mothers (310, 311), causing stress and family discord linked to PPD. These findings militate for PPD intervention efforts focusing on providing support for working mothers.

Our finding that maternal mortality predicts 19% of the cross-national variation in PPD prevalence can be interpreted in several ways. First, suicide linked to mental illness is a major cause of maternal mortality in many countries (1, 2). However, maternal mortality is also a reliable proxy of poor access to medical care, consistent with our finding that higher rates of infant mortality and low birth weight also predicted higher national PPD prevalence. The relationship between maternal mortality and PPD is likely bidirectional, with PPD driving maternal mortality rates and poor healthcare driving both maternal mortality and PPD risk. Therefore, efforts to improve either of these outcomes are likely to evince spillover benefits improving the other. Relatedly, high total fertility rates predicted elevated PPD prevalence, suggesting that improved access to contraception associated with healthcare services may also reduce national PPD prevalence.

## Limitations

Several methodological limitations should be considered when interpreting the results of this meta-analysis and meta-regression. First, clinical interviews are the gold standard for PPD diagnosis, whereas our analysis focused on a widely used self-report measure. Self-report measures tend to yield higher estimates of PPD than clinical interviews, therefore, our estimates are likely higher than if we had focused on interview methods (6). However, given the serious consequences of PPD, we felt it was better to potentially overestimate than to underestimate national prevalence. Second, several countries had few studies (e.g., Finland, Mexico, and Nepal), rendering those national estimates less reliable relative to countries where the bulk of PPD research has been done (e.g., the United States, the United Kingdom, and Australia). Finally, many potential predictors of cross-national PPD prevalence were beyond the scope of this study ranging from degree of cultural collectivism to rates of vitamin D deficiency (311–313). We hope that the data set provided in this study will allow future researchers to uncover additional structural, cultural and health predictors of cross-national variation in PPD prevalence.

## Conclusion

In sum, our findings reveal that the global prevalence of PPD is both higher and more variable than previously thought, and that wealth inequality, maternal-child health indexes, and employment patterns explain most of the cross-national variation. Creating meaningful improvements in these areas presents enormous social challenges, yet the potential benefits of reducing PPD for mothers, families, and infants are equally great.

## Author Contributions

JH-H conceptualized the research questions, conducted the analysis, wrote the paper, and approved this manuscript. TC-H and IA helped to compile the data set, write the manuscript, and approved this manuscript.

## Conflict of Interest Statement

The authors declare that the research was conducted in the absence of any commercial or financial relationships that could be construed as a potential conflict of interest.

## Appendix

### Boolean Search Information

CINAHL

Boolean search that yields 142 records:

(IN edinburgh postnatal depression scale) AND (AB (postpartum depression OR postnatal depression)) AND (AB (incidence OR prevalence))

With the additional limiters:

(1) Narrow to 1985–2015
(2) Narrow to English articles only
(3) Narrow to Humans only
(4) Narrow to Females only

Permanent Link: http://libproxy.chapman.edu/login?url=https://search.ebscohost.com/login.aspx?direct=true&AuthType=ip,uid,cookie,url&db=rzh&bquery=(IN+edinburgh+postnatal+depression+scale)+AND+(AB+(postpartum+depression+OR+postnatal+depression))+AND+(AB+(incidence+OR+prevalence))&cli0=DT1&clv0=198501-201512&cli1=CT3&clv1=Female&cli2=LA99&clv2=eng&type=1&site=ehost-live

PsychInfo

Boolean search that yields 236 records:

AB (incidence or prevalence) AND AB (postnatal depression or postpartum depression) AND TM edinburgh postnatal depression scale

With the additional limiters:

(1) Narrow to 1985–2015
(2) Narrow to English articles only
(3) Narrow to females only

http://libproxy.chapman.edu/login?url=https://search.ebscohost.com/login.aspx?direct=true&AuthType=ip,uid,cookie,url&db=psyh&bquery=(AB+(incidence+OR+prevalence))+AND+(AB+(postnatal+depression+OR+postpartum+depression))+AND+(TM+edinburgh+postnatal+depression+scale)&cli0=PY&clv0=198501-201512&cli1=LA1&clv1=Y&cli2=PO2&clv2=Female&type=1&site=ehost-live

PubMed

Boolean search yields 338 records:

((“postpartum depression”[All Fields] OR “postnatal depression”[All Fields]) AND “prevalence”[All Fields]) AND “edinburgh postnatal depression scale”[All Fields] AND ((“1985/01/01”[PDAT]:?“2015/12/31”[PDAT]) AND “humans”[MeSH Terms])
