# Supplementary figures and images for: Economic and Health Predictors of National Postpartum Depression Prevalence: A Systematic Review, Meta-analysis, and Meta-Regression of 291 Studies from 56 Countries
Source: Front Psychiatry. 2018 Feb 1;8:248. doi: 10.3389/fpsyt.2017.00248 (PMC5799244; doi:10.3389/fpsyt.2017.00248)

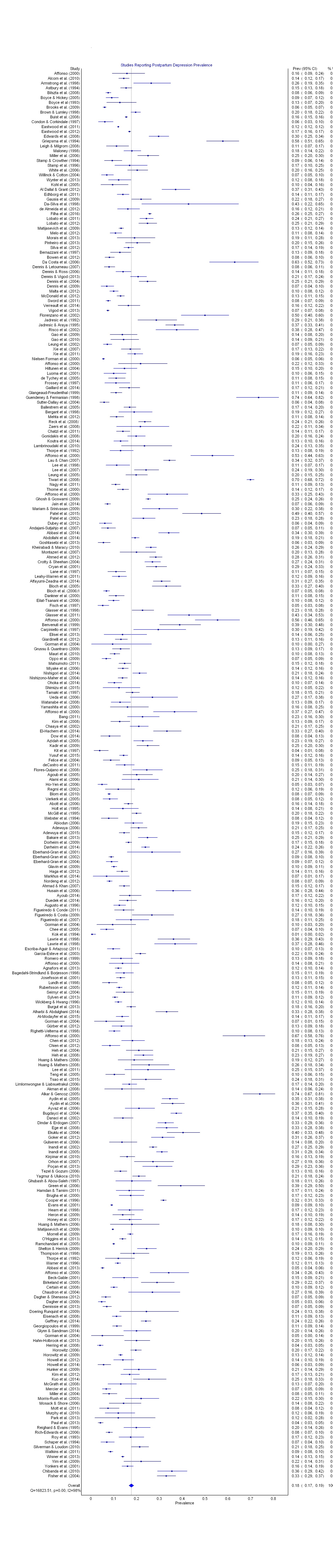

Supplement: Supplementary file 2 [file Image_1.jpg]
